# Big five and higher-order personality traits matter for climate-risk perception among moroccan farmers

**DOI:** 10.1038/s41598-026-57036-w

**Published:** 2026-06-11

**Authors:** Perez L. Kemeni Kambiet, Adan L. Martinez-Cruz, Tarik Chfadi, Ernest L. Molua, Kaouthar Saddiqui, Victor Ongoma

**Affiliations:** 1 International Water Research Institute, Mohammed VI University Polytechnic, Benguerir, Morocco; 2 Center for Independent Development Research, Buea, Cameroon; 3 Department of Forest, Environmental and Resource Economics, Swedish University of Agricultural Sciences, Uppsala, Sweden; 4 Department of Agricultural Economics and Agribusiness, University of Buea, Buea, Cameroon

**Keywords:** Climate change, Vulnerable communities, Climate-risk perception, Big five traits, Meta-personality traits, Environmental social sciences, Psychology, Psychology

## Abstract

Climate change risk perception is increasingly recognized as an important antecedent of climate-related engagement, yet its psychological foundations remain underexplored in vulnerable, non-WEIRD contexts. This study examines associations between personality traits and affective climate-risk perception (denoting climate-related worry), among cereal farmers in Morocco. Drawing on the Big five and higher-order meta-trait personality framework, we model affective climate-risk perception as a function of both individual traits and their composite configurations. Using cross-sectional data from cereal-farming households, we estimate these associations through different analytical specifications. Results show that personality traits are significantly associated with affective climate-risk perception. At the trait level, Emotional Stability is consistently linked to lower levels of reported climate-related worry, whereas Extraversion and Openness are associated with higher worry. The findings suggest that engagement-oriented and information-seeking dispositions correspond with heightened emotional sensitivity to climate threats. At the meta-trait level, Meta-Plasticity is positively associated with affective climate-risk perception, whereas Meta-Stability is associated with lower worry. Exploratory composite configurations further reveal a non-linear, U-shaped association for a Social Harmony orientation. This indicates that moderate social embeddedness may coincide with lower concern, while very high levels are associated with increased affective climate-risk perception. Overall, the findings do not imply causal effects on behavior but demonstrate that perceived affective climate-risk is patterned along stable psychological dimensions, even under similar exposure conditions. By integrating these personality traits into affect-based climate-risk frameworks, this study highlights the role of psychological heterogeneity in shaping farmers’ responses to climate-risks and underscores the need for more targeted adaptation, climate communication, and resilience-building strategies in vulnerable agricultural communities.

**Supplementary Information:**

The online version contains supplementary material available at 10.1038/s41598-026-57036-w.

## Introduction

The growing frequency and intensity of climate extremes, together with global recognition of climate change as a threat to sustainable development, has prompted a shift in climate policy design^1,2^. Shaped by a reorientation in how stakeholders and policymakers understand and frame climate challenges, this new perspective positions individuals not only as contributors to global warming but as essential agents in its mitigation^1,2^. Through sustainable choices such as responsible consumption, waste management, and resource use, individuals can collectively reduce carbon emissions and help build a resilient economic future^3–5^. Related estimates suggest that coordinated behavioral shifts could cut emissions by up to 7.2 tons of CO₂-equivalent per capita annually^6^. Further, these actions often align with broader ecological restoration efforts, such as reforestation, which simultaneously combat climate change, enhance biodiversity, and improve ecosystem services^7^.

Such potential has spurred extensive research into how to promote sustainable behaviors at national and regional levels^8–10^. Among these efforts, climate change perception has emerged as a critical domain. Climate change perception is fundamentally a cognitive-behavioral process in which individuals’ beliefs, motivations, emotions, personality traits, and environmental experiences shape their interpretation of climate risks and their willingness to undertake adaptation actions^11–15^. As such, studying perception offers location-specific insights into vulnerabilities, preferences, and behavioral tendencies^12,13^ which are essential for crafting inclusive climate policies and effective communication strategies^14^. Yet, despite the recognized importance of perception, there remains an unsettling debate on the most effective ways to leverage it. While studies agree that direct observation of climate impacts can drive behavioral change, the psychological factors that shape perception are less understood^15^.

Indeed, evidence suggest that climate change risk perception is formed at the intersection of physical observation and internal psychological models^14–16^. Even so, the psychological component of perception is generally under-studied^9,14^. Most notably, the affective dimension—the level of worry, concern and emotions an individual feels—remains a frequently overlooked aspect of risk perception, despite being an equally potent driver of action as cognitive assessments of risk^2,9,14–16^. Moreover, both cognitive and affective psychological factors are important in explaining the significant heterogeneity in responses observed within communities that share similar physical environment^9,17,18^. To account for these psychological factors while understanding group differences in responses, studies have turned to individual personality traits as a basis for mapping these differences^19,20^.

Although many psychological factors can explain behavioral heterogeneity, the five-factor personality traits stand as a foundational starting point^19–22^. Unlike other factors, the five-factor model of personality—Openness, Conscientiousness, Extraversion, Agreeableness, and Neuroticism (hereafter Big five traits)—is well-grounded in social, behavioral, and neuroscientific literature as representing stable, fundamental tendencies in cognition, emotion, and behavior^23–25^. However, the application of this framework to climate action reveals several critical gaps. Current studies^3–5^ often link personality directly to environmental behaviors, thereby bypassing the mediating role of risk perception. In doing so, existing works not only treat perception as an implicit afterthought but also largely neglect its critical affective dimension. Additionally, studies on sustainable choices are overwhelmingly concentrated in developed (Western, Educated, Industrial and Democratic-WEIRD) countries, even though sustainable policies also hold significant benefits for developing (non-WEIRD) nations^26^.

Further, the literature indicates that Big five traits cluster into higher-order meta-traits of Plasticity and Stability, which are associated with broader life outcomes in developed contexts^27–29^. Yet, limited evidence exists on how these meta-traits operate under conditions of high climate vulnerability, or how they relate to climate-related outcomes such as affective risk perception.

In this study, we address these gaps by examining the association between Big five traits and their higher-order meta-traits with affective climate-risk perception. Employing different analytical specifications on a sample of 331 cereal-farming households from the Marrakesh-Safi area of Morocco, we show that both Big five traits and higher-order configurations are significantly associated with affective climate-risk perception. Our findings highlight both expected and counter-intuitive associations, including the potentially buffering role of Emotional Stability and the positive association of Extraversion and Openness. The meta-traits of Plasticity and Stability reveal strong associations that suggest a psychological trade-off between emotional regulation and information-driven urgency. Similarly, the Social Harmony exploratory composite shows a non-linear (U-shaped) association with affective climate-risk perception, indicating the complexity that underlies psycho-affective associations.

By revealing these associations beyond the Big five traits, our results offer novel insights for designing targeted behavioral interventions, including mitigation, adaptation, and communication strategies. Our contributions also extend the climate risk perception literature in three ways. First, we foreground the affective dimension of climate risk perception, which is often overlooked in favor of cognitive assessments, and construct a robust measure of affective climate-risk perception that captures the emotional dimension of perceived climate-risks. Second, we provide one of the first empirical analyses linking Big five traits to affective climate-risk perception in a high-vulnerability, non-WEIRD context. Third, we complement and extend prior work on behavioral paradoxes in climate-risk perception and adaptation in Morocco^30^, situating psycho-affective associations as a critical dimension of climate-risk perception. Lastly, we move beyond trait-level analysis to examine how personality traits cluster into higher-order meta-traits of Plasticity and Stability, thereby offering new insights into how personality dispositions relate to the emotional dimension of climate risk perception under conditions of climate vulnerability.

## Personality traits and affective risk perception: a conceptual framework

Climate change perception and adaptation may be conceptualized as a multi-dimensional process shaped by cognition, personality, behavioral motivation, environmental opportunities, and socio-economic risk structures^13–16^. An integrated theoretical framework combining psychological theories on personality and climate-risk models may provide a strong basis for understanding: climate perception, adaptation behavior, resilience, and long-term sustainability outcomes. However, although widely conceptualized as central to explaining emotional regulation, goal setting and adaptive behaviors, few theories have explicitly posited how fundamental personality traits relate to climate-risk perception^31–34^. To bridge this gap, we draw on two complementary sets of psychological theories. The first set (comprising Construal Level Theory, Affordance Theory, and Reinforcement Sensitivity Theory) explains how individuals translate external stimuli into internal risk perception and motivation^35–40^. The second set (Cybernetic Big Five Theory and personality neuroscience) explains how Big five traits provide the underlying individual differences and reaction to risks^27,28^.

The first set of theories addresses how physical and mental stimuli are conceived and acted upon. Construal Level Theory suggests that individuals interpret events based on their perceived psychological distance; distant threats are construed abstractly, reducing the perceived need for immediate action^35,36^. Affordance Theory conceptualizes perception as a guide for action^37–39,41^. For a farmer, the environment is rich with affordances e.g., a new drought-resistant seed ‘affords’ the possibility of a stable harvest, while a dry riverbed ‘affords’ the perception of an immediate threat. Building on these ideas, Reinforcement Sensitivity Theory (RST) elaborates how such perceptions modulate behavior^38,39^. The RST posits that perceptions of threat or opportunity activate distinct motivational subsystems: the Behavioral Inhibition System (BIS), which responds to punishment and threat (generating worry), and the Behavioral Activation System (BAS), which responds to reward and opportunity^37^. Critically, the sensitivity of these BIS/BAS subsystems is known to be strongly associated with foundational personality traits, particularly Emotional Stability and Extraversion^35,36,42^.

**Fig. 1:**
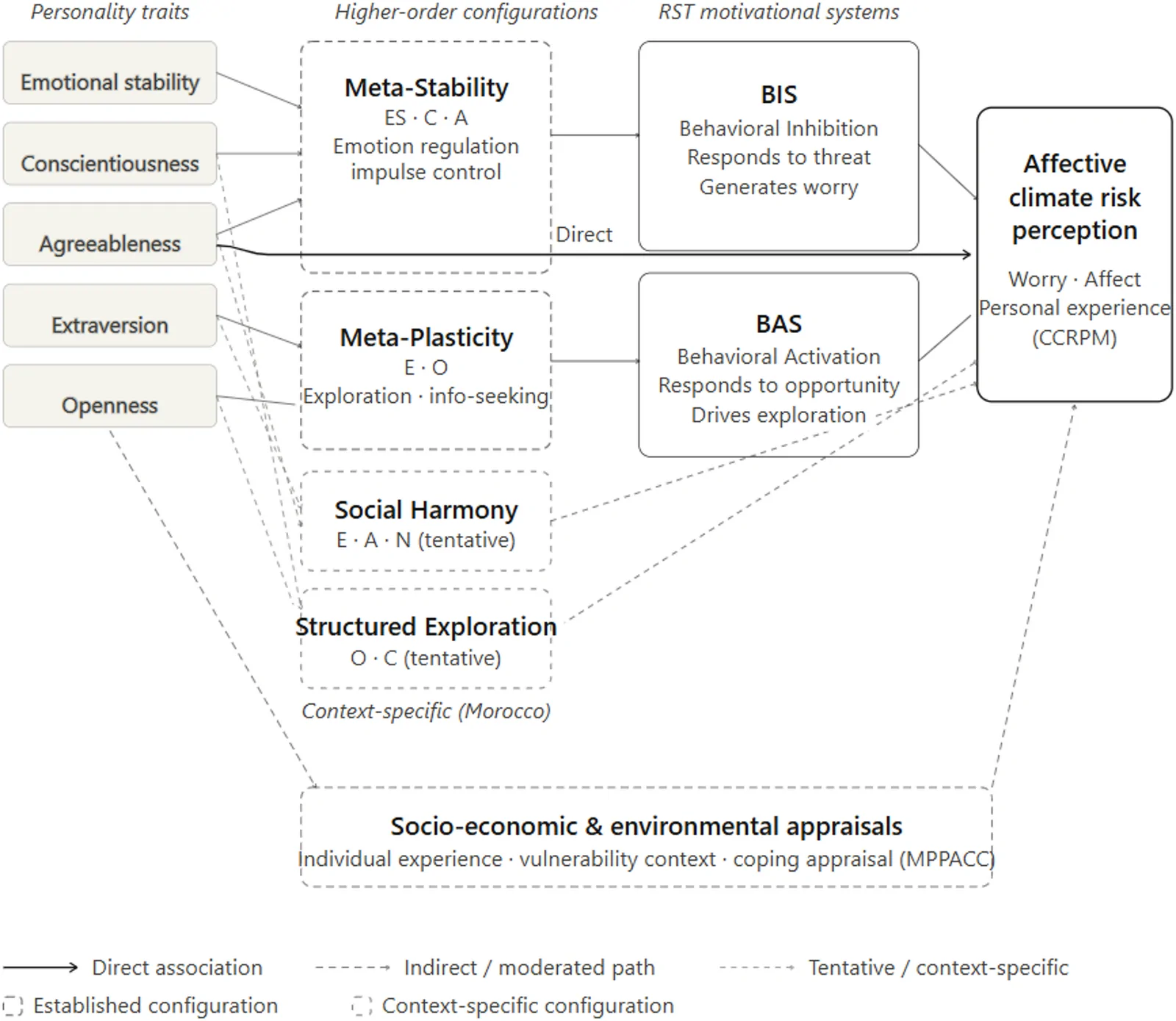
Personality traits and Affective climate-risk perception. Source: Authors’ Conceptualization.

The second set reinforces the notion that Big five traits are the foundations of individual construal, association, and action in the world. Complementarily, Cybernetic Big Five Theory frames personality meta-traits as systems that guide how individuals pursue goals amidst uncertainty^27,28,43^. Meta-Stability (composed of Emotional Stability, Agreeableness, and Conscientiousness) relates to emotional regulation and impulse control, aligning with the function of the BIS^35–37,42,43^. Meta-Plasticity (composed of Extraversion and Openness) reflects tendencies toward exploration and information-seeking, mirroring the BAS function^24,27^. These higher-ordered configurations have also been shown to predict broader life outcomes^27–29^, but their relevance under conditions of climate vulnerability remains underexplored. Furthermore, studies^31,43–46^ suggest contexts can trigger the unique higher-ordered configurations of Big five traits that are useful for survival in specific environmental contexts. Adding to these and given the paradoxes of farmers’ climate responses in Morocco^30^, we assess the existence of such contextual configuration as Social Harmony (a tentative composite involving Extraversion, Agreeableness, and Neuroticism), Structured Exploration (an exploratory composite of Openness and Conscientiousness). Building on these insights, Fig. 1 represents the plausible context-sensitive direct and indirect links between personality traits and farmers affective risk perceptions in Morocco.

Additionally, blending these theoretical and contextual sensitivities to our framework advances and complements two leading climate-risk and adaptation theories, the Climate Change Risk Perception Model (CCRPM) and the Model of Private Proactive Adaptation to Climate Change (MPPACC). Van der linden’s CCRPM emphasizes the role of affect, personal experience, and worry in shaping risk perception^15,16^. Grothmann and Patt’s MPPACC highlights how risk perception interacts with coping appraisals to drive adaptation^47^. As presented in Fig. 1, we introduce personality traits and higher-order meta-traits as antecedent dispositions that explain how the affective dimension of risk perception is construed given individual experiences, socio-economic and environmental appraisals. As such, we extend CCRPM by specifying likely sources of individual differences in affective responses, and we enrich MPPACC by clarifying how personality traits may condition emotional appraisals of climate threats.

Overall, the Construal Level Theory, Affordance Theory, Reinforcement Sensitivity Theory, Cybernetic Big Five Theory, and the climate-risk/adaptation models collectively show that climate adaptation is not only an economic or technological process, but also cognitive, behavioral, psychological, institutional, personality-driven and socially embedded. Taken together, this integrated framework suggests that personality traits matter for affective climate-risk perception. The psycho-affective link may be direct, through Big five traits, or indirect, through established meta-traits of Meta-Plasticity and Meta-Stability and contextual higher-order configuration such as Social Harmony and Structured Exploration. By highlighting these associations, the framework offers context‑sensitive approach to understanding emotional responses to climate change in vulnerable, non‑WEIRD settings.

## Methodology

### Study area: climate vulnerability and agricultural context in the Marrakech-Safi region of Morocco

This study is conducted in the Marrakesh-Safi region (MSR) of west-central Morocco. The area provides a natural laboratory for investigating psychological responses to climate change. Geographically, it covers 38,167 km² and is comprised of the Marrakesh prefecture and seven provinces, bordered by the Atlantic Ocean to the west and the High Atlas Mountains to the south^48,49^. The region’s climate is predominantly semi-arid, characterized by highly variable and low rainfall, with nearly half the area receiving less than 300 mm annually^50^. This inherent water scarcity, combined with a diverse topography of plains, plateaus, and mountains, makes MSR exceptionally susceptible to the impacts of climate change^48,51^.

The primary livelihood for the region’s rural and communal population is agriculture, which has been prone to climate extremes in the last two decades. Persistent droughts, rising temperatures, and declining precipitation have led to critical groundwater depletion and significant losses in agricultural productivity^48,51^. Cereal farming, particularly of rainfed crops like wheat and barley, is central to both subsistence and income for the region’s 96,612 farmers^49,52,53^. Despite frequent and substantial crop losses due to climate shifts, these farmers have demonstrated remarkable resilience, consistently adapting their cropping calendars (typically running from November to March) to the onset of seasonal rains^54,55^.

The government has set up an institutional support system across the region’s 7 provinces and their associated communes to support agricultural activities. Notably, the Direction Régionale de l’Agriculture (DRA) and the Office Régional De Mise En Valeur Agricole (ORMVA) oversee resource management across MSR’s provinces via their provincial counterparts (Direction Provincial de l’Agriculture - DPA)^53,56^. At the local level, the Centre de Conseil Agricole (CCA) serves as the primary extension service, implementing national strategies like the Generation Green 2020–2030 and distributing subsidized planting materials to farmers at the start of each season^52,56^. This institutional interaction, combined with the shared environmental adversity, creates a unique setting to investigate how stable individual psychological traits relate to varying levels of perception and affective climate responses among farmers who face similar objective risks.

**Fig. 2:**
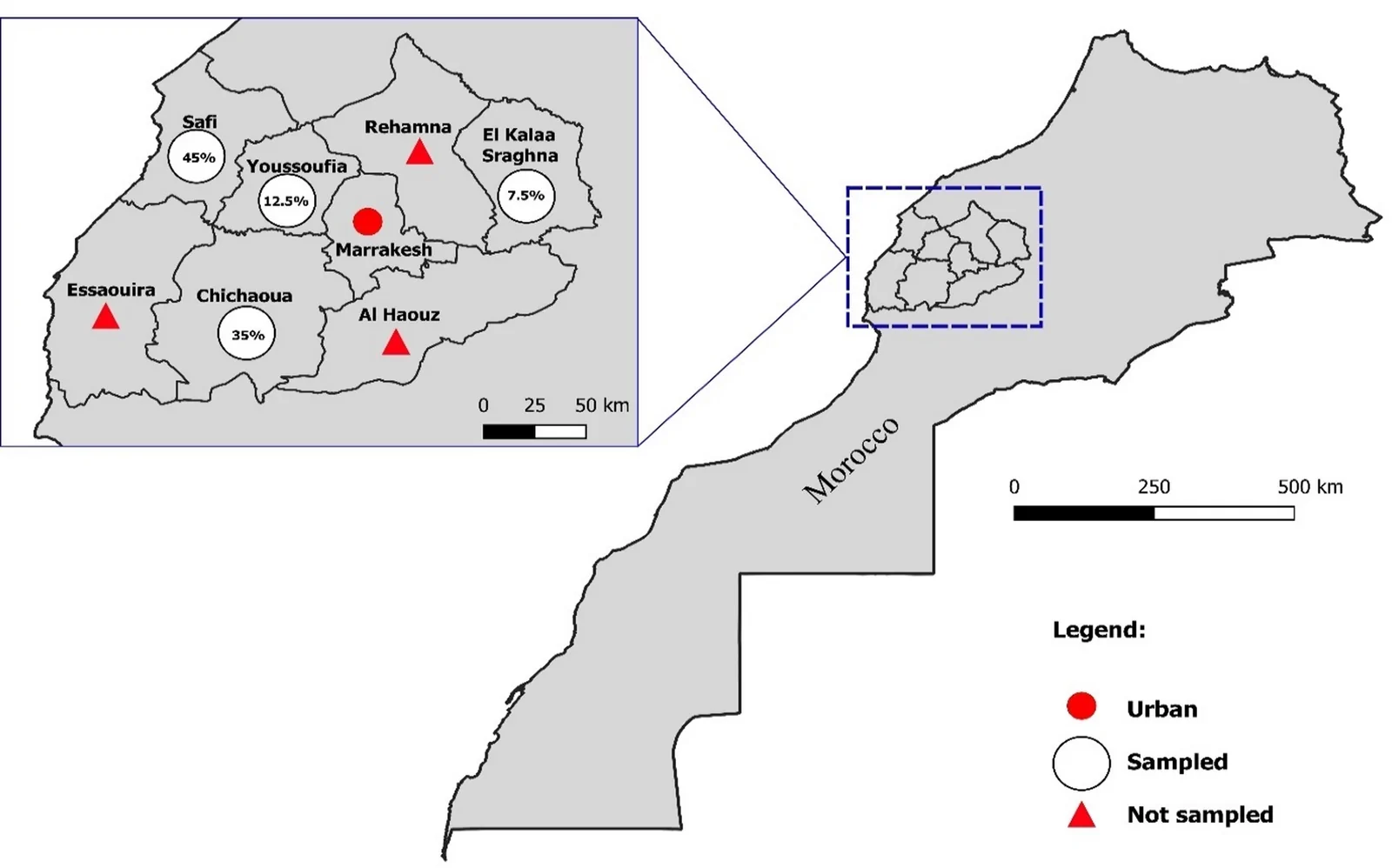
Map of the Marrakesh-Safi region with sampled and unsampled areas. Source: Authors’ Conceptualization.

### Data collection and sampling

This study relies on field survey data that was collected between November 2024 and March 2025 across four provinces within Morocco’s Marrakesh-Safi region (Fig. 2). A multi-stage sampling design was employed to ensure representation across the diverse microclimates and topographies relevant to rainfed cereal production in the region.

The first stage involved the purposive selection of four provinces from the seven in the region. In consultation with experts from key agricultural institutions (DRA, ORMVA, and DPA), the choice enumeration provinces were based on the topographical features of each province as well as the concentration of cereal farming households in the area (Fig. 2). In the second stage, we selected enumeration communes in each enumeration province, and a sampling quota was determined for each selected commune based on the most recent official census of active cereal farmers^53^.

In the third stage, we conducted computer-assisted personal interview (CAPI) with a final sample representing 400 cereal-farming households (Fig. 2). This sampling process was facilitated by the cooperation of local agricultural extension centers (Centre de Conseil Agricole—CCA). Precisely, the CCA provided the list of farmers that participated during the 2024/2025 subsidized input distribution campaign to guide sampling in enumeration communes. All sections of the survey questionnaire were translated into the main local language (Darija), pre-tested and administered by trained enumerators in the local languages (Darija and Tamazight). The questionnaire collected data on a range of topics including household demographics, production practices, social capital, affective climate-risk perception, and personality traits. The Big five personality traits were assessed using the validated 20-item International Personality Item Pool (IPIP)^29^.

All participants offered verbal consent and were informed of their right to withdraw or skip questions. A number of respondents chose to omit some personality, psychological and asset modules items, citing personal sensitivity. Consequently, the final analytical sample for this study comprises the 331 respondents who provided complete data for all items that are relevant for assessing the association of personality traits to affective climate-risk perception when socio-economic and contextual experiences are considered (Specifically, seven respondents selected “Don’t Know” in at least one of the 20-item IPIP module; we therefore considered only complete cases in constructing composite Big five traits. In addition, 30 respondents in the initial survey skipped household and location-specific items—including physical assets owned, residence time, number of rooms, and number of income earners—due to perceived sensitivity. Assisted by the associated Centre de Conseil Agricole (CCA), mobile follow-up was later conducted to complement these missing socio-economic data for the 30 farmers; however, the present study is based on the balanced sample derived from the initial survey. As such, our analytic sample represents 331 cereal-farming households containing complete personality, affective climate-risk perception, household, physical, and farm characteristic information). The sample selection formula and calculations are given in Appendix S0.

### Measurement of personality traits

Personality traits are measured using the 20-item International Personality Item Pool (IPIP-20), a validated short form of the Big five inventory that has been widely applied in diverse cultural and field settings^19,20,29^. Each Big five trait (Openness, Conscientiousness, Extraversion, Agreeableness, and Neuroticism) was assessed using four items rated on a five-point Likert scale (1 = “strongly disagree” to 5 = “strongly agree”), including both direct and reverse-coded items.

 After appropriate scoring, confirmatory and exploratory factor analysis (CFA/EFA) was conducted on the items to assess validity and reliability. Confirmatory factor analysis of the IPIP-20 items demonstrated consistent and significant factor loading for all items (*p* < 0.001), with moderate overall fit indices (CFI = 0.84; RMSEA = 0.12; Appendix S1a, Table S1a1). While these indices fall slightly below conventional thresholds, they are consistent with (and frequently exceed) fit levels reported in personality research conducted in rural, low-literacy, and non-WEIRD populations^26,44,45^. Importantly, all scales demonstrated acceptable to excellent internal consistency (α = 0.65–0.93; Appendix S1a, Table S1a2), supporting their suitability for subsequent analysis. Given the consistency of CFA/EFA, these items were averaged to produce composite Big five trait measures (Table 1). A summary of reliability coefficients is presented in Table 1, with full CFA results in Appendix S1a. For consistency, Emotional Stability was subsequently operationalized as reverse Neuroticism.

**Table 1:** Summary Statistics for Big five personality traits.

Trait	Cronbach’s Alpha	Std. Loading Range	Interpretation
Openness	0.79	0.68–0.87	Robust loadings
Conscientiousness	0.65	0.55–0.83	Moderate reliability, acceptable in field settings
Extraversion	0.77	0.63–0.88	Robust loadings
Agreeableness	0.93	0.93–0.94	Excellent reliability
Neuroticism	0.83	0.60–0.91	High reliability
CFA Fit Indices	χ²(82) = 529.62, *p* < 0.001; CFI = 0.84; TLI = 0.80; RMSEA = 0.128; SRMR = 0.118		Moderate overall fit

### Measurement of meta-trait configurations

While the Big five traits capture foundational dispositions, our conceptual framework posits that individuals perceive environmental risk through broader Big five trait complexes that are often captured as higher-order personality configurations^24,27,57^. Moreover, strands of literature in (non)western societies point towards the notion of traits association as essential for explaining individual behaviors^31,42,44–46^. This study leans on such evidence to argue that the communal context of Morocco might motivate the association of Big five traits in ways that are linked to farmers’ perception of climate-related affect. To empirically operationalize these complexes while minimizing multicollinearity, we constructed four meta-trait configurations. Notably two established meta-traits (Meta-Stability and Meta-Plasticity) alongside two context-specific configurations composites (Structured Exploration and Social Harmony). These composites are treated explicitly as exploratory configurations, not as new personality traits, and are intended to assess whether socially embedded or norm-constrained trait combinations correspond to non-linear or context-dependent patterns of affective climate-risk perception.

Given the mathematical complexity and convergence challenges of second-order factor models in cross-cultural field data, we employed Principal Component Analysis (PCA) to construct these higher-order variables. This approach is considered best practice in personality research in heterogeneous populations^46^. For each configuration, PCA was performed on the relevant subset of Big five traits, and components were retained based on Kaiser’s criterion (Eigenvalue > 1). In all cases, only the first principal component met this criterion (Appendix S1b, Table S1b1), capturing between 49.6% and 55.5% of the variance.

The retained first components therefore represent the dominant psychological composites underlying each configuration. In Table 2 Factor loadings confirm that Meta-Stability reflects emotional regulation and self-control (Agreeableness, Conscientiousness, Emotional Stability), Meta-Plasticity captures exploration and engagement (Openness, Extraversion), Structured Exploration reflects context-aware exploratory orientation (Openness and Conscientiousness), and Social Harmony captures emotionally regulated social embeddedness (Extraversion, Agreeableness, Emotional Stability) (Appendix S1b, Table S1b2). Table 1 summarizes the conceptual definitions and trait compositions of the four resulting higher-order personality variables.

**Table 2:** Summary Statistics for Meta‑trait configurations.

Configuration	Originating Traits	Loading (PC1)	Eigenvalue	% Var Explained
Meta-Stability	Agreeableness	0.761	1.489	49.6%
	Conscientiousness	0.515		
	Emotional Stability	0.803		
Meta-Plasticity	Extraversion	0.736	1.084	54.2%
	Openness	0.736		
Structured Exploration	Openness	−0.732	1.072	53.6%
	Conscientiousness	0.732		
Social Harmony	Extraversion	0.706	1.666	55.5%
	Agreeableness	0.804		
	Emotional Stability	0.722		

**Notes**: According to Kaiser’s criterion, acceptance is reserved for all Eigen > 1.

### Measurement of affective climate-risk perception

 Affective climate-risk perception is operationalized through a constructed composite that measures farmers climate-related worry using 5-item self-assessment questions. To guide the design of a 5-item tool, we leveraged past studies^4,5,15,16^ which noted that farmers better understand affect when it directly underscores views about the impact of climate-related shocks to their livelihoods. In this light, both general and specific self-assessment questions are used to capture climate-related worry as lived in cereal-farming households. To reflect construal-level processes, the measure begins with a general item assessing overall climate-related worry, followed by four domain-specific items capturing worry related to core livelihood dimensions: farm yields, livestock health, household income, and household health (Appendix S1; Appendix S2, Table S2a). Respondents rated each item on a five-point Likert scale ranging from 1 (“not at all worried”) to 5 (“extremely worried”).

In line with psychometric standard for construct validation, we conducted confirmatory and exploratory reliability analysis. Our analysis indicated a high internal consistency (Cronbach’s α = 0.89; Appendix S2, Table S2c). Exploratory factor analysis equally confirmed the unidimensional composite, with the dominant first component (Eigenvalue = 4.37) explaining 87% of variance (Appendix S2, Table S2b), supported by adequate KMO values (0.691–0.854) and a significant Bartlett’s test (*p* = 0.044). Confirmatory factor analysis further validated the one-factor solution, with excellent fit indices (χ²(4) = 14.05, *p* = 0.007; CFI = 0.993; TLI = 0.982; SRMR = 0.026). Based on the convergence of CFA/EFA results, we extracted scores from the first principal component to construct a continuous measure for Affective climate-risk perception, which assesses the level of worry for each farmer. A summary of reliability and fit statistics is presented in Table 3, while full item loadings and diagnostics are reported in Appendix S2.

**Table 3:** Summary Statistics for affective climate-risk perception.

Statistics	Value/Range	Interpretation
Cronbach’s Alpha	0.89	Excellent internal consistency
KMO (per item range)	0.691–0.854	Sampling adequacy acceptable to excellent
Bartlett’s Test (p-value)	0.044	Significant correlations among items
Eigenvalue (PC1)	4.37	Strong first factor
Variance Explained (PC1)	87%	Substantial proportion of variance
CFA Fit Indices	χ²(4) = 14.05, *p* = 0.007; CFI = 0.993; TLI = 0.982; RMSEA = 0.087 (90% CI: 0.040–0.138); SRMR = 0.026	Good overall fit

### Empirical estimation strategy

We examine the association between personality traits and affective climate-risk perception in two sequential steps^58^. The first step estimates the direct association of each Big five traits with affective climate-risk perception. The second explores whether higher-order meta-trait configurations including Meta-Stability, Meta-Plasticity, Structured Exploration, and Social Harmony reveal additional contextual dynamics that individual trait-level analysis could miss. All models are Ordinary Least Squares (OLS) estimations with heteroscedasticity-consistent robust standard errors to ensure reliable inferences^58,59^.

Precisely, we begin with a linear specification that regresses affective climate-risk on the Big five traits as given in Eq. 1.

1 $$\:{y}_{AR,i}={\beta\:}_{0}+\:{X}_{i,\:traits}{\beta\:}_{traits}+\:{X}_{i,control}{\beta\:}_{controls}+\:{\epsilon\:}_{i}\:$$

where $\:{y}_{AR,i}$ denotes affective climate-risk perception–a proxy of the intensity of climate climate-related worry expressed by farmer * i*. $\:{\beta\:}_{0}$ is the intercept term, $\:{X}_{i,\:traits}$ denotes the Big five traits for farmer * i* while $\:{\beta\:}_{traits}$ is the coefficient of interest and it captures the association of Big five traits to the intensity of climate-related worry expressed by the farmer. To improve the precision of our estimation, we also control household and farm specific factors. $\:{X}_{i,control}$ underscore other relevant covariates including socio-economic, demographic, and farm-specific characteristics and their associated coefficients $\:{\beta\:}_{controls}$. All unobservable factors are represented by the stochastic random term $\:{\epsilon\:}_{i}$.

In the second stage, we examine whether higher‑order personality configurations are associated with affective climate‑risk perception. This stage is also designed to explore the possible nuanced relationships that arise from context-sensitive synergy and interaction between Big five traits. Given that the preliminary analyses revealed a significant quadratic (U-shaped) relationship for the exploration of Social Harmony higher-order configuration, we specify a regression model of affective climate-risk against higher-order composite configuration which explicitly incorporates a non-linear Social Harmony term, specified as in Eq. 2:

2 $$\:{y}_{AR,i}={\beta\:}_{0}+\:{X}_{i,\:S}{\beta\:}_{S}+{X}_{i,\:P}{\beta\:}_{P}+\:{X}_{i,\:SE}{\beta\:}_{SE}+{X}_{i,\:SH}{\beta\:}_{SH}+{X}_{i,\:SH}^{2}{\beta\:}_{SH}+\:{X}_{i,control}{\beta\:}_{controls}+\:{\epsilon\:}_{i}$$

where $\:{y}_{AR,i}$ denotes the affective climate-risk perception of farmer * i*, while $\:{X}_{i,\:S,\:P,\:SE,\:SH}\:$ are the linear terms representing higher-order meta-traits–Meta-Stability, Meta-Plasticity, Structured Exploration and Social Harmony. $\:{X}_{i,\:SH}^{2}$ is the squared term for the Social Harmony composite configuration and their associated $\:\beta\:$ are the coefficients of interest. We also control household and farm specific covariates $\:{X}_{i,control}$ and their associated coefficients$\:\:{\beta\:}_{controls}$. Unobservable factors are captured in the stochastic random term $\:{\epsilon\:}_{i}$.

Finally, to assess robustness and parsimony, we compared three specifications: a base model with only personality predictors, a full model including socio-demographic and farm-specific covariates, and a reduced model identified through backward stepwise selection guided by the Akaike Information Criterion (AIC). It is important to note that, the results are interpreted as average associations and the stepwise AIC procedure is used only as a robustness check to confirm the stability of results, not as the primary inferential strategy^58,59^. The staged design allows us to compare trait-level associations with those observed for higher-order personality configurations, and to assess whether composite personality orientations capture heterogeneity in affective climate-risk perception beyond individual traits.

### Ethics declaration

This study was conducted in accordance with the ethical standards of Mohammed VI Polytechnic University (UM6P), which adheres to the principles outlined in the Declaration of Helsinki. Ethical approval was obtained from UM6P through the International Water Research Institute (IWRI) prior to the commencement of data collection. The research involved questionnaire-based surveys with human participants and was approved in line with UM6P research ethics and integrity guidelines. At the time of approval (2023/2024), the approving body did not assign a formal numeric ethics approval reference. Additional authorization for field survey administration was secured from the *Centre de Conseil Agricole* (CCA) of the respective enumeration provinces. All participants provided informed consent verbally prior to participation (This study used verbal informed consent since the Ethics Review Body recognized that forcing a written signature protocol within these specific rural farming communities introduces socio-cultural barriers, elevates participant apprehension, and causes severe selection bias due to varying literacy levels). Participation was voluntary, and respondents were assured of confidentiality and anonymity in data handling and reporting. No personally identifiable information was collected, and all data were analyzed in aggregate form.

## Results and discussions

### Descriptive statistics

Table 4 presents a summary of the key variables used in this study. Regarding our outcome variable, the five risk perception items, measured on a 1-to-5 Likert scale, show mean scores ranging from 2.14 to 2.78. Farmers expressed the highest levels of concern for climate risks that affect their crop yields (mean = 2.78) and their community in general (mean = 2.68). Conversely, they reported lower average concern for household income and health. This may reflect the perceived immediacy of agricultural impacts (on food security) versus other livelihood aspects. The affective climate-risk perception measure is a continuous variable centered by construction, with a mean of approximately zero (min = −3.38, max = 1.89).

The Big five traits revealed a distinct personality profile for this farming community. Notably, farmers reported very high levels of Conscientiousness (mean = 3.97), Agreeableness (mean = 3.93), and Extraversion (mean = 3.82). These above-average scores likely reflect the social and practical demands of farming in a vulnerable, communal context, where diligence, planning, and strong social networks are vital for adaptation and accessing support^60,61^. In contrast, scores were lower for Openness (mean = 2.85) and farmers reported high Emotional Stability (mean = 3.00). The lower Openness may reflect the value placed on conformity to group ideals over novel ideas in some communal settings. The high Emotional Stability could indicate a psychological self-selection, where only farmers with a certain resilience to stress have remained in such a challenging and volatile profession.

The higher order meta-traits configurations indicate centrality with mean around zero, which is ideal for their use in linear regression models. More revealing than the means, however, are their ranges of variation. Meta-Stability and Social Harmony exhibited the narrowest ranges, potentially reflecting a baseline level of social cohesion and stability that is necessary for life in a communal society^62^. Conversely, the Structured Exploration and Meta-Plasticity showed much wider variations^63^. This intuitively suggests that while a certain level of social stability is a shared norm, the predispositions toward exploring new ideas, innovation and external engagement are where the greatest individual differences lie, even within a uniform community^63^.

For the socio-demographic and farm-specific characteristics we considered respondent household and farm specific variables. Farmers have an average age that falls within the 59 to 79 age range. Farmers’ age however varies from a minimum of 20 to a maximum of 80 years. This reflects a common trend of low youth engagement in agriculture observed across Morocco and other developing nations^54^. Formal education levels are modest, with an average of 4.5 years of schooling, consistent with findings on rural educational attainment in Morocco^64,65^.

The household structure is indicative of the region’s communal lifestyle. The average residency time in the community is approximately 50 years, suggesting a strong attachment to place of birth and family, a tendency noted by research on community-based action^61^. Households are large, with an average of 6 members and on average 2 income-earners per household. Regarding climate exposure, farmers reported experiencing an average of four major extreme weather events over the last two decades. This high level of direct exposure, combined with their long residency, indicates that most farmers possess a deep, longitudinal understanding of the changes in their local environment.

Farm characteristics reveal that the average land holding is 17.6 hectares. Most farmers described their land as having a plain topography (66%) and being of high fertility (73%). Farmers typically resided in brick-and-mortar homes with an average of 4 rooms and held a moderate number of productive and livestock assets. As suggested by foundational models of adaptive capacity^66^, the reliance on these assets is a key feature of rural livelihoods, serving as a critical source of liquidity and a buffer against the shocks and uncertainties inherent to their environment. Appendix S3 presents a correlation matrix of these variables.

**Table 4:** Summary of farm household and farm characteristics.

Variable	Variable Type	Min.	Mean	Max.
Climate-related worry variables				
General household wellbeing	Categorical	1.00	2.68	5.00
Crop Yield	Categorical	1.00	2.78	5.00
Household Health	Categorical	1.00	1.68	5.00
Household Income	Categorical	1.00	2.14	5.00
Livestock Health	Categorical	1.00	2.02	5.00
Perceived affective climate-risk	Continuous	−3.38	0.00	1.89
Personality Traits				
Openness	Continuous	1.75	2.85	4.00
Conscientiousness	Continuous	2.75	3.93	4.75
Extraversion	Continuous	2.00	3.82	5.00
Agreeableness	Continuous	2.50	3.97	5.00
Emotional Stability	Continuous	1.25	3.00	4.00
Neuroticism	Continuous	1.25	2.25	4.00
Meta-Trait Configurations				
Meta-Stability	Continuous	−3.06	0.04	2.14
Meta-Plasticity	Continuous	−3.84	0.08	2.63
Structured Explore	Continuous	−4.23	0.07	2.21
Social Harmony	Continuous	−3.55	−0.01	2.24
Household Characteristics				
Age	Categorical	1.00	2.22	4.00
Age (20–39)	Dummy 1	0	0.21	1
Age (40–59)	Dummy 2	0	0.42	1
Age (60–79)	Dummy 3	0	0.34	1
Age (> 80)	Dummy 4	0	0.03	1
Level of Education	Continuous	0.00	4.52	18.00
Residence Time	Continuous	1.00	50.92	96.00
Household Size	Continuous	1.00	6.00	25.00
Number of Income Earners	Continuous	1.00	1.63	10.00
Climate Exposure Intensity	Continuous	0.00	4.98	18.00
Farm Characteristics & Assets				
Topography [Plain = 1]	Binary	0.00	0.66	1.00
Fertility [High = 1]	Binary	0.00	0.73	1.00
Land Size	Continuous	1.00	17.63	400.00
Number of Rooms	Discrete	1.00	4.24	15.00
Productive Assets	Continuous	0.00	9.10	16.00
Livestock Assets	Discrete	0.00	2.21	6.00

### Estimates of empirical analysis

The econometric results are presented in two parts, each summarizing three models that are detailed in Appendices S4 and S5. In both parts, model [A] presents baseline OLS estimates without controls, model [B] incorporates the full set of socio-economic controls, and model [C] applies a parsimonious backward stepwise procedure as an internal robustness check. Part one examines the direct association between affective climate-risk perception and the Big five traits, while Part two explores the indirect relationship through higher-order meta-traits.

### Big five traits and affective climate-risk perception

**Fig. 3:**
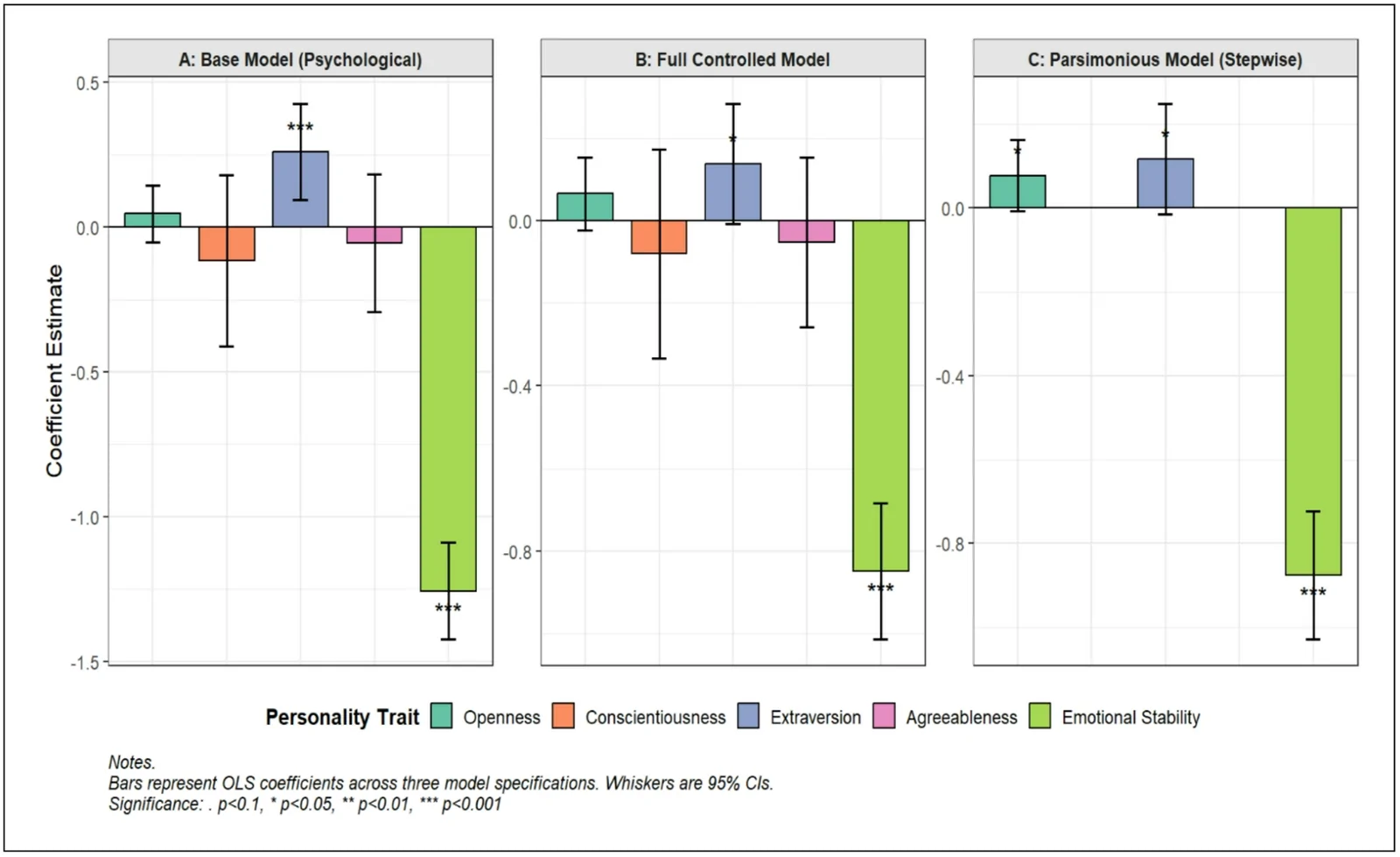
Estimates of the Interaction of Big five traits with the level of affective climate-risk perceived. Source: Authors Construction from Regression Estimations| Notes. Dependent variable is the affective climate-risk perceived (denoting farmers worry). Robust standard errors are in parentheses. Significance levels: * *p* < 0.1, ** *p* < 0.05, *** *p* < 0.01.

Figure 3 presents the estimated associations of Big five traits to the level of affective climate-risk perceived by cereal farmers. Results show that personality traits relate to affective risk perception in distinct ways, with Emotional Stability and Extraversion exhibiting the most consistent and statistically significant associations across specifications. This is expected since empirical studies suggest that these traits relate individual emotions and our measure of affective climate-risk perception reflects climate-related worry. These findings are also consistent with psychological theory suggesting that both Emotional Stability and Extraversion may intrinsically be linked to the emotional regulatory systems, specifically the Behavioral Inhibition and Activation Systems (BIS/BAS), that shape responses to potential threats and rewards^35,36,42^.

Looking at the direction of these relationships, estimates depict expected and counter-intuitive associations. Conscientiousness and Emotional Stability negatively relate to affective climate-risk perception suggesting that farmers who are Conscientiousness coupled with higher Emotional Stability are less likely to be worried about climate-related risks. As evidenced in^3,21^, stronger Emotional Stability reduces the perceived negative feelings that are related to risk and uncertainty. Given that Conscientiousness has been evidenced as strong predictor for self-regulation^19,20,24,27^, a plausible interpretation may be that self-regulated and emotionally stable individuals are less reactive to negative affect, enabling them to remain less worried amidst climate uncertainty. This aligns with a broad body of research highlighting the role of self-regulation in maintaining goal-driven behaviors under stress^24,27,57^. In our case, the persistence of farmers in a climate-sensitive agricultural system may suggest a selection effect for a baseline level of self-regulation and emotional stability^60^.

Additionally, the negative association observed for Agreeableness, a trait often linked to empathy and prosocial orientation, appears at first glance to contradict parts of the existing literature^19–22,57^. However, this pattern may also reflect the role of communal resilience within the study context. According to^30^, in highly communal societies like Morocco, strong social support networks can serve as critical buffers against environmental shocks. Related studies^62,69^ corroborate the suggestion that individuals with high Agreeableness may be more embedded in prosocial networks and therefore perceive a lower personal level of climate-related worry, owing to greater confidence in collective capacity to cope with adversity.

In contrast, Openness and Extraversion show positive associations. While other studies suggest that abstract thinking (Openness) and social connectedness (Extraversion) would facilitate access to innovative adaptation methods, through a corresponding link to lower perception of affective climate‑risk^24,27^, the Moroccan context suggests the opposite. Although contrary, these results resonate with a selected strand of literature in communal societies where high Openness have been linked to increased anxiety especially when new ideas conflict with communal norms, while Extraversion may expand social obligations alongside resources^67^. An alternatively aligned explanation comes from the literature on personality traits and information-seeking behaviors^42,43,48,63,70^. Studies of this view argue that both traits foster information‑seeking, which can heighten awareness of climate threats and, in turn, amplify affective climate‑risk perception^43,68^. Taken together, these counter-intuitive estimates draw towards^43,68^ and likely reflect the communal case where the central role of Openness and Extraversion in information-seeking correlates with a heightened sense of urgency regarding climate threats.

Finally, the introduction of socio-economic controls in Model [B] and the parsimonious specification Model [C] confirms the robustness of Emotional Stability and Extraversion. The absence of Agreeableness and Conscientiousness indicates that their associations are context-dependent and masked by socio-demographic variables. Also, Openness re-emerges with marginal significance, possibly underscoring the complex association of personality to socio-cultural environment. Such results reinforce the case^30^ surrounding the complexity and behavioral paradoxes that underlie perception and climate adaptation attitudes in Morocco. We explore such context emerging interactions and their relation to higher-order meta-traits in the next section.

### Higher-ordered meta-personality traits, and affective climate-risk perception

**Fig. 4:**
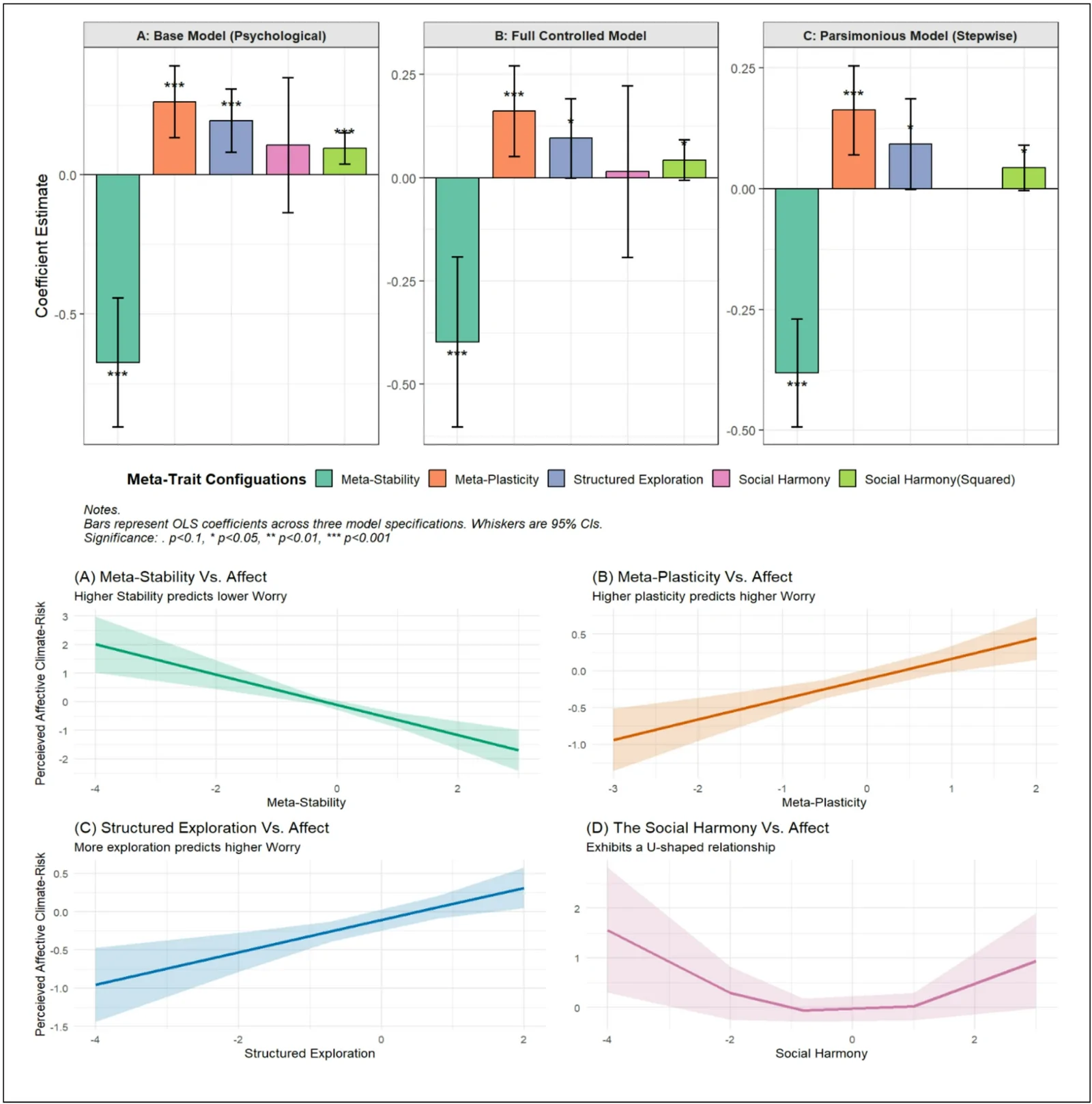
Estimated link between Meta-trait configuration and perceived affective climate-risk. Source: Authors Construction from Regression Estimations. Notes. Dependent variable is the affective climate-risk perceived (denoting farmers worry). Robust standard errors are in parentheses. Significance levels: * *p* < 0.1, ** *p* < 0.05, *** *p* < 0.01.

Building on the Big five results, we extend the analysis to higher-order personality configurations to examine whether meta-traits offer context nuanced insights for understanding the relationship between personality traits and affective climate-risk responses among farmers. This approach also allows us to explore the coherence of these contextual associations with existing personality, risk and adaptation theories as laid out in the conceptual framework (Fig. 1). Figure 4 represents the estimated associations between higher-order meta-traits and affective climate-risk perception. In the baseline specification (Model A), all four meta-traits exhibit statistically significant associations albeit the contrast between Meta-Stability and Meta-Plasticity. Meta-Stability, a composite of Emotional Stability, Agreeableness, and Conscientiousness, is negatively associated with affective climate-risk perception, indicating lower reported climate-related worry among individuals characterized by emotional regulation, impulse control, and social order. This pattern aligns with evidence linking emotional stability and self-regulation to reduced negative affect under risk and uncertainty^19–21,33,60,71^. It is also consistent with personality-neuroscientific accounts that associate Meta-Stability with the regulation of threat-related emotional responses through inhibitory control systems^24,27,42^.

Conversely, Meta-Plasticity, combining Openness and Extraversion, shows a positive association with affective climate-risk perception (β = 0.263, *p* < 0.01). This suggests that exploratory, socially engaged, and cognitively flexible dispositions tend to co-occur with higher affective worry regarding climate threats. Rather than contradicting the role of Meta-Plasticity in adaptive engagement, this association resonates with work linking Openness and Extraversion to information-seeking, environmental awareness, and heightened sensitivity to novel or uncertain stimuli^20,22,43,71^. Collectively, the opposing signs of Meta-Stability and Meta-Plasticity indicate a psychological tension in affective climate-risk perception, wherein emotional containment and self-regulation correspond to lower worry, while exploration and engagement correspond to heightened worry. Though associational, this contrast complements affect-centered climate-risk models by organizing affective responses into coherent personality orientations^15,16^.

The exploratory higher-order composites further illustrate the contextual nature of these dynamics and corroborate studies that noted the emergence of trait complexes as essential for navigating specific environments^44–46^. Structured Exploration, combining Openness and Conscientiousness, exhibits a positive association (β = 0.195, *p* < 0.01). This suggests that disciplined exploration may be accompanied by increased affective (climate-related worry) in environments where innovation unfolds under uncertainty or normative constraints. This notion is consistent with research highlighting anxiety responses when openness to novelty interacts with structured social or institutional settings^24,42,67^. Social Harmony, composed of Extraversion, Agreeableness, and Neuroticism, displays a non-linear (U-shaped) association, whereby moderate social integration corresponds to lower affective climate-risk perception, while very high integration is associated with increased worry.

This non-linear pattern is particularly salient in communal and interdependent settings. Prior studies emphasize that strong social networks can buffer vulnerability by facilitating mutual support and collective coping^30,61,62,69^. At the same time, dense social integration may amplify shared emotional responses through social transmission of worry and collective stress, especially under common environmental threats^57,67^. The observed U-shaped association for Social Harmony thus complements existing literature^30^ by suggesting that communal embeddedness may both attenuate and intensify affective climate-risk perception, depending on the degree of social integration.

Robustness checks reinforce these interpretations. With the inclusion of socio-economic controls in Model [B], the associations for Meta-Stability (β = − 0.397, *p* < 0.01) and Meta-Plasticity (β = 0.162, *p* < 0.01) remain statistically significant but attenuated, indicating partial overlap with socio-demographic characteristics such as assets, household structure, and exposure^17,60,71^. The parsimonious specification in Model [C] confirms the central role of these meta-traits: Meta-Stability continues to show the strongest negative association (β = − 0.381, *p* < 0.01), while Meta-Plasticity remains positively associated (β = 0.163, *p* < 0.01). Structured Exploration retains marginal significance, and the non-linear Social Harmony relationship persists (squared term: β = 0.044, *p* < 0.1), as illustrated in lower panel (D) of Fig. 4.

Overall, the meta-trait analysis complements and extends the Big five literature by demonstrating that affective climate-risk perception is associated not only to individual traits, but also broader personality orientations toward emotional regulation, exploration, and social embeddedness. In doing so, these findings contribute to reconciling mixed trait-level evidence^19–22^ and integrate personality structure into affect-based climate-risk frameworks^15,16,47^, particularly in vulnerable, non-WEIRD, agricultural contexts^26^. Hence, behavioral response constitutes a critical foundation for climate change risk coping and adaptation, as psychological distance may influence climate risk perception, environmental affordances could determine perceived opportunities for adaptive action, and motivational systems may shape adaptive decision-making. Together, these cognitive, behavioral, and environmental mechanisms underpin resilience formation, and adaptive capacity enhancement. Such insights are thus essential for guiding the design of context sensitive, climate policies and communication strategies.

### Conclusion and policy implications

This study examines how personality traits and higher-order personality configurations are associated with affective climate-risk perception among cereal farmers in a climate-vulnerable, non-WEIRD context. Applying different estimation approaches on cross-sectional data, we identified patterns linking individual personality differences to heterogeneity in expressed affective climate-risk perception.

At the trait level, Emotional Stability and Extraversion displayed the most consistent associations with affective climate-risk perception across specifications, highlighting the relevance of stable emotional regulation and engagement-oriented dispositions. Importantly, these associations remain consistent after accounting for socio-economic, demographic, and farm-level characteristics, indicating that affective climate-risk perception is not solely a function of exposure or material conditions.

Moving beyond individual traits, the analysis showed that higher-order personality configurations, notably the meta-traits i.e. Meta-Stability and Meta-Plasticity, help organize these trait-level associations into coherent psychological orientations. Meta-Stability was consistently associated with lower levels of expressed climate-related worry, whereas Meta-Plasticity was associated with heightened concern. Exploratory composite configurations further suggested that socially embedded and exploratory orientations may relate to affective climate-risk perception in context-specific and non-linear ways, underscoring the importance of social and cultural settings in shaping emotional responses to climate threats.

Noteworthy, these findings do not imply that personality determines climate-related behavior or adaptation outcomes. Rather, they indicate the existence of psycho-affective link that is inherent to farmers experiencing climate-related shocks. By situating personality structure within affect-based climate-risk frameworks, this study complements existing models by clarifying sources of heterogeneity in emotional responses to climate change, particularly in smallholder agricultural systems where exposure is widespread, but climate-related worry varies considerably.

These results carry important implications for climate communication and adaptation policy, while remaining mindful of the study’s observational nature. First, the prominence of affective climate-risk perception suggests that uniform climate messaging may not resonate equally across individuals. For instance, farmers characterized by high emotional stability may report lower worry even under similar exposure conditions, whereas those with engagement-oriented dispositions may express heightened concern. Climate services, extension systems, and early-warning communication may therefore benefit from differentiated framing strategies that acknowledge heterogeneity in affective responses without equating concern with adaptive capacity.

Second, the relevance of higher-order personality configurations underscores the value of moving beyond single traits when designing community-level interventions. In highly communal settings, strong social embeddedness may buffer perceived affective climate-risk up to a point but also has the potential to amplify shared worry. This finding suggests that community-based adaptation and information-sharing initiatives should be sensitive to individual and collective emotional dynamics, especially during periods of heightened climate stress.

Third, by focusing on affective climate-risk perception, this study highlights affective climate-risk perception (worry) as an upstream psychological component of adaptation processes. While adaptive behavior is not examined directly, affective risk perception plays a critical role in shaping attention, information uptake, and engagement with adaptation options. Policies aimed at promoting climate resilience may therefore be more effective when they address emotional responses alongside informational and structural constraints.

Some limitations warrant consideration. First, the cross-sectional design limits the study’s inference and does not allow assessment of how perceived affective climate-risk translates into behavior over time. Second, the analysis relies on self-reported measures, which may be subject to reporting biases. Furthermore, while the internal consistency coefficients of the Big five scales are acceptable to strong, the first-order CFA fit statistics remain moderate. While this is common and conceptually defensible within rural, non-WEIRD populations characterized by varying literacy levels, it naturally necessitates caution. These statistical boundaries place inherent limits on the certitude with which more elaborate higher-order configurations can be interpreted on top of the first-order trait structure, highlighting the critical need for cross-cultural psychometric refinement in similar empirical settings. Likewise, the wider conceptual models that support the associations in this study are yet to be assessed explicitly, hence necessitating the need for caution. Also, the contextual nature of our exploratory and standard higher-order configurations may hinder the generalization of our findings. As such, future research combining longitudinal designs, behavioral outcomes, and richer psychosocial measures would be particularly valuable for testing how perception of affective climate-risk interacts with coping appraisals and actual adaptation behavior. Such work would further clarify the role of personality in climate vulnerability and resilience, especially in under-represented, non-WEIRD contexts. Another noteworthy limitation is the absence of formal pre-registration of hypotheses and analytical procedures. Although the empirical strategy and robustness checks were transparently reported, the lack of pre-registration means that the analyses cannot be fully distinguished from exploratory work. This reduces the reproducibility and open science alignment of our study. Future research can therefore adopt pre-registration protocols and make data and code openly available to strengthen transparency, replicability, and adherence to evolving standards of scientific integrity. Nonetheless, our results provide a valuable starting point for bridging psychological dispositions with climate adaptation policy, offering a more nuanced understanding of how farmers perceive and act upon climate-risks for plausible long-term resilience, and sustainable development outcomes.

## Supplementary Information

Below is the link to the electronic supplementary material.

Supplementary Material 1

## Data Availability

Data will be made available upon request and approval by the International Water Research Institute, Mohammed VI Polytechnic University Benguerir, Morocco.
