# Supplementary material for: Big five and higher-order personality traits matter for climate-risk perception among moroccan farmers
Source: Sci Rep. 2026 Jun 11;16:26580. doi: 10.1038/s41598-026-57036-w (PMC13503745; doi:10.1038/s41598-026-57036-w)
Supplement: Supplementary file 1 [file 41598_2026_57036_MOESM1_ESM.docx]

**APPENDIX**

**Big Five and Higher-Order Personality Traits Matter for Climate-Risk Perception Among Moroccan Farmers**

**Appendix S0.** Sample Size Calculation

The minimum required sample size was calculated using Cochran’s formula with finite population correction (FPC):

$$n=\frac{z^{2}\cdot p\cdot(1-p)}{e^{2}}\text{ }/\text{ }\left[ 1 + \frac{z^{2}\cdot p\cdot(1-p)}{e^{2}\cdot N} \right]$$

Where: $z=1.96$(for 95% confidence level); $p=0.5$(proportion, chosen to maximize variability); $e=0.05$(margin of error, 5%); $N=96,612$(population size of the study area)

**Calculation :**

$$n=\frac{{1.96}^{2}\cdot0.5\cdot(1-0.5)}{{0.05}^{2}}\text{ }/\text{ }\left[ 1 + \frac{{1.96}^{2}\cdot0.5\cdot(1-0.5)}{{0.05}^{2}\cdot96,612} \right]$$

$$n=\frac{384.16}{1.004}\approx382.64$$

Thus, the minimum required sample size is approximately 383 respondents.

**Distribution of our Final Sample**

To ensure robustness and account for potential non-response or incomplete data, we targeted 400 cereal-farming households. The sample was proportionally allocated across the four provinces based on their population shares:

**Table S0.** Distribution of Sampled Cereal farming households

| Province | Population Share (%) | Sample Size (n) |
| --- | --- | --- |
| Kelaa des Sraghna | 7.5 | 30 |
| Youssoufia | 12.5 | 50 |
| Safi | 45 | 180 |
| Chichaoua | 35 | 140 |
| Total | **100** | **400** |

**Appendix S1a:** Psychometric Assessment of the IPIP-20 items

**Confirmatory Factor Analysis (CFA)**

Table S1a1 summarizes the first-order CFA maximum likelihood (ML) for the IPIP-20 Big Five items. The estimates suggest a normal convergence with significant factor loadings for all items after 51 iterations. Fit indices indicate a moderate fit: χ² (82) = 529.62, p < 0.001; CFI = 0.84; TLI = 0.80; RMSEA = 0.118 (90% CI: 0.118–0.139); SRMR = 0.118. Chi‑square test is significant, but the comparative fit indices fall slightly below conventional thresholds (CFI/TLI ≥ 0.90), and RMSEA exceeds the recommended cut‑off (<0.08). While the global fit indices (CFI = 0.84; RMSEA = 0.12) fall slightly under the traditional 0.90 threshold. These indices are also consistent with (and often exceed) typical fit levels reported in personality research conducted in non-WEIRD (Western, Educated, Industrialized, Rich, and Democratic), rural, and non-student populations (Gurven et al., 2013; Laajaj et al., 2019). Given that all items loaded significantly on their respective factors and the internal consistency was robust, the scales were deemed suitable for analysis.

**Table S1a1.** Confirmatory Factor Analysis Results for IPIP-20 Personality Items

| Latent Variable | Item | Std. Loading | SE | z‑value | Sig. |
| --- | --- | --- | --- | --- | --- |
| Openness | OP_1 | 0.77 | 0.02 | 15.74 | *** |
|  | OP_2_rev | 0.86 | 0.04 | 18.56 | *** |
|  | OP_3_rev | 0.87 | 0.05 | 19.99 | *** |
|  | OP_4_rev | 0.68 | 0.03 | 13.21 | *** |
| Conscientiousness | CON_1 | 0.83 | 0.02 | 14.79 | *** |
|  | CON_2 | 0.55 | 0.05 | 9.49 | *** |
|  | CON_3 | 0.67 | 0.02 | 11.94 | *** |
|  | CON_4 | 0.60 | 0.03 | 10.84 | *** |
| Extraversion | ET_1_rev | 0.63 | 0.05 | 11.86 | *** |
|  | ET_2_rev | 0.65 | 0.04 | 12.21 | *** |
|  | ET_3 | 0.88 | 0.03 | 18.08 | *** |
|  | ET_4 | 0.69 | 0.03 | 13.14 | *** |
| Agreeableness | AX_1 | 0.63 | 0.05 | 11.65 | *** |
|  | AX_2 | 0.62 | 0.05 | 11.23 | *** |
|  | AX_2_rev | 0.94 | 0.02 | 24.09 | *** |
|  | AX_4_rev | 0.93 | 0.02 | 24.09 | *** |
| Neuroticism | NE_1 | 0.91 | 0.04 | 20.24 | *** |
|  | NE_2_rev | 0.60 | 0.03 | 11.60 | *** |
|  | NE_3 | 0.88 | 0.04 | 19.13 | *** |
|  | NE_4_rev | 0.61 | 0.02 | 11.83 | *** |
| Model fit indices: | χ²(82) = 529.62, p < 0.001 | CFI = 0.84 | TLI = 0.80 | RMSEA = 0.128; (90% CI: 0.118–0.139) | SRMR = 0.118 |

**Notes:** ***p < 0.001; **p < 0.01; *p < 0.05; All loadings are statistically significant at p < 0.001; Cronbach’s alpha values are reported for each IPIP-20 item.

**Big Five Trait Reliability Analysis (Exploratory Factor Analysis)**

Cronbach’s Alpha (α) is further evaluated for trait composites to ensure reliability and the internal consistency of each underlying Big Five trait. As shown in Table S1a2, the scales underscore good to excellent reliability. Agreeableness (α = 0.93), and Neuroticism (α = 0.83) showed high reliability as well as Openness and Extraversion. Emotional stability is computed as reverse Neuroticism and Conscientiousness (α = 0.65) is slightly lower but considered acceptable, especially with respect to the diversity of the items and the nature of survey data.

**Table S1a2:** Factor Loadings and Reliability Coefficients

| Construct | Items | Std. Loading | Cronbach’s Alpha (α) |
| --- | --- | --- | --- |
| Openness | 4 Items | 0.68 – 0.87 | 0.79 |
| Conscientiousness | 4 Items | 0.55 – 0.83 | 0.65 |
| Extraversion | 4 Items | 0.63 – 0.88 | 0.77 |
| Agreeableness | 4 Items | 0.93 – 0.94 | 0.93 |
| Neuroticism | 4 Items | 0.60 – 0.91 | 0.83 |

**Appendix S1b:** Evaluating Unidimensional Meta-Personality Configurations

Due to the mathematical complexity and the possible over-identification of second-order models, Meta-traits (Stability and Plasticity) and contextual composites (Social Harmony and Structured Exploration) were constructed using Principal Component Analysis (PCA). This approach is preferred in cross-cultural research when structural equation models fail to converge due to localized variations in trait covariance (Benet-Martínez & John, 1998).

To ensure transparency in the construction of our higher-order personality variables, we present the results for the first two principal components for each configuration (Table S1b1). We applied the Kaiser Criterion, which mandates that only components with an Eigenvalue greater than 1 should be retained for score construction.

In all cases, only the first component (PC1) met this threshold. For instance, in the Social Harmony configuration, PC1 yielded a strong Eigenvalue of 1.66, while PC2 fell significantly below the threshold at 0.75. Similarly, for Structured Exploration, PC1 (Eigenvalue = 1.07) was retained while PC2 (0.92) was rejected.

While PC2 in the Plasticity and Structured Exploration configurations explains a significant portion of variance (nearly 46%), it fails to reach the cutoff (Eigenvalue of 1) indicating that it does not capture a dimension distinct from the first. By focusing only on PC1, *we ensure that the 'Structure Exploration' and 'Social Harmony' configurations represent context-sensitive psychological construct. Specifically, the shared variance between the traits*. As shown in Table S1b2, *PC1 for Structured Exploration underscore the exploratory composite that captures the 'orientation of farmers towards abstraction that fits within communal norms'* by highlighting the tension between Openness and Conscientiousness, whereas PC2 reflects their sum, losing the context-aware 'Exploration' logic central to the realities within communal communities.

**Table S1b1:** PCA Component Selection and Kaiser Criterion Justification

| Higher-Order Configuration | Component | Eigenvalue | % Variance | Cumulative % | Kaiser Decision |
| --- | --- | --- | --- | --- | --- |
| Meta-Stability | **PC1** | **1.489** | **49.6%** | **49.6%** | **Accepted** |
|  | PC2 | 0.902 | 30.1% | 79.7% | Rejected (Eigen < 1) |
| Meta-Plasticity | **PC1** | **1.084** | **54.2%** | **54.2%** | **Accepted** |
|  | PC2 | 0.916 | 45.8% | 100.0% | Rejected (Eigen < 1) |
| Structured Exploration | **PC1** | **1.072** | **53.6%** | **53.6%** | **Accepted** |
|  | PC2 | 0.928 | 46.4% | 100.0% | Rejected (Eigen < 1) |
| Social Harmony | **PC1** | **1.666** | **55.5%** | **55.5%** | **Accepted** |
|  | PC2 | 0.757 | 25.2% | 80.8% | Rejected (Eigen < 1) |

**Notes:** According to Kaiser’s criterion, acceptance is reserved for all Eigen > 1

**Table S1b2: Factor Loadings for Primary (PC1) and Secondary (PC2) Components**

| Configuration | Trait | PC1 Loading | PC2 Loading | PC1 Interpretation |
| --- | --- | --- | --- | --- |
| Meta-Stability | Agreeableness | 0.761 | -0.389 | **General Stability:** |
|  | Conscientiousness | 0.515 | 0.849 | Balanced self-regulation. |
|  | Emotional Stability | 0.803 | -0.176 |  |
| Meta-Plasticity | Extraversion | 0.736 | -0.677 | **General Plasticity:** |
|  | Openness | 0.736 | 0.677 | Exploration & Engagement. |
| Structured Exploration | Openness | **-0.732** | 0.681 | **Orientation towards Context-aware Exploration:** |
|  | Conscientiousness | **0.732** | 0.681 | Context-aware Exploration & Engagement. |
| Social Harmony | Extraversion | 0.706 | 0.639 | **Orientation towards Communal Harmony:** |
|  | Agreeableness | 0.804 | --- | Prosocial Emotional Regulation. |
|  | Emotional Stability | 0.722 | -0.590 |  |

**Appendix S2.** Measurement of Affective Climate-Risk Perception

The Measure of Affective Climate-Risk is constructed from the itemized questions of Table S2a and consists of 5 questions. Each question evaluates the level of climate-related worry that a farmer expresses with respect to aspects of their community and household. The Likert scale varies from One (01—Not at all worried) through two and three (02 Slightly Worried; 03—Worried) to Four and Five (4—Very Worried and 5—Extremely Worried). The measure is obtained through initial Confirmatory Factor Analysis (CFA) and Exploratory Factor Analysis validation as seen in Table S2b and Table S2c

**Table S2a. 5-Item Affective Climate-Risk Perception Self-Assessment question(s)**

| **Code** | **Itemized self-assessment questions** | **Perception dimension** |
| --- | --- | --- |
| Concern_cc_effect | Based on your experiences in the community, how worried are you about the effect of climate change in general? | Affordance |
| Concern_cc_effect1 | Based on your experiences in the community, how worried are you about the effect of climate change on your farm? | Construal |
| Concern_cc_effect2 | Based on your experiences in the community, how worried are you about the effect of climate change on household health? | Construal |
| Concern_cc_effect3 | Based on your experiences in the community, how worried are you about the effect of climate change on household income? | Construal |
| Cocncern_cc_effect4 | Based on your experiences in the community, how worried are you about the effect of climate change on livestock health? | Construal |

**Affective Climate-Risk Perception Confirmatory Factor Analysis (CFA)**

The Affective Risk (Concern) Index exhibited excellent fit indices: χ²(4) = 14.05, p < 0.01; CFI = 0.99; TLI = 0.98; and SRMR = 0.026. This confirms that the concern items effectively represent a single latent dimension of affective risk perception.

**Table S2b.** Confirmatory Factor Analysis Results for Affective Climate‑Risk Perception

| Latent Variable | Item Code | Std. Loading | SE | z‑value | Sig. | KMO |
| --- | --- | --- | --- | --- | --- | --- |
| Affective Climate‑Risk Perception | Concern_cc_effect | 1.38 | 0.08 | 17.51 | *** | 0.821 |
|  | Concern_cc_effect1 | 1.04 | 0.08 | 13.80 | *** | 0.691 |
|  | Concern_cc_effect2 | 1.11 | 0.09 | 13.11 | *** | 0.854 |
|  | Concern_cc_effect3 | 1.22 | 0.09 | 13.96 | *** | 0.690 |
|  | Cocncern_cc_effect4 | 1.69 | 0.08 | 22.27 | *** | 0.803 |
| Model fit indices: | χ²(4) = 14.05, p = 0.007 | CFI = 0.993 | TLI = 0.982 | RMSEA = 0.087 (90% CI: 0.040–0.138) | SRMR = 0.026 |  |

**Notes.** ***p < 0.001; **p < 0.01; *p < 0.05; All loadings are statistically significant at p < 0.001; KMO values range from (0.691–0.854) suggesting a strong association of the items that are used for constructing our measure of Affective Climate-Risk Perception.

**Affective Climate-Risk Perception Exploratory Factor Analysis (EFA)**

Table S2c and d represent results of confirmatory tests and the exploratory factor analysis. Cronbach Alpha is above the 0.7 cutoff range and Bartlett’s test (p = 0.044) indicates significance at 95% level of significance further confirming sampling adequacy and item correlations (Table S2c). Moreover, the factor analysis shows a single dominant factor (Eigenvalue = 4.37) explaining 87% of observed variance. Table S2d demonstrates that the other components contribute minimally and their variance falls far below the kaiser criterion’s threshold of 1. This distribution indicates that our PCA derived component is relevant and appreciably captures the affective climate-risk construct that is being measured. The convergence of EFA and CFA on a unidimensional construct for Affective Climate‑Risk Perception internally validates our measure as a reliable measure of climate‑related worry.

**Table S2c. Summary Statistics for Construct Validation**

| Statistic | Value / Range | Interpretation |
| --- | --- | --- |
| Cronbach’s Alpha | 0.89 | Excellent internal consistency |
| Bartlett’s Test (p‑value) | 0.044 | Significant correlations among items, suitable for factor analysis |
| Eigenvalue (PC1) | 4.37 | Strong first factor |
| Variance Explained (PC1) | 87% | Substantial proportion of variance captured by one |

**Notes:** According to Kaiser’s criterion, acceptance is reserved for all Eigen > 1

**Table S2d.** PCA all components of Concern Index

| **Variable** | **Component 1** | **Component 2** | **Component 3** | **Component 4** | **Component 5** |
| --- | --- | --- | --- | --- | --- |
| **Component Loadings** | **Concern Index** | **Not extracted** | **Not extracted** | **Not extracted** | **Not extracted** |
| General household wellbeing | 0.84 | 0.05 | -0.02 | 0.01 | 0.03 |
| Crop Yield | 0.87 | 0.03 | 0.01 | -0.04 | 0.01 |
| Household Health | 0.69 | 0.07 | -0.05 | 0.02 | 0.04 |
| Household Income | 0.87 | -0.02 | 0.03 | 0.01 | -0.02 |
| Livestock Health | 0.90 | 0.04 | 0.02 | -0.03 | 0.01 |
| **Variance Explained** |  |  |  |  |  |
| Eigenvalue | 4.37 | 0.28 | 0.20 | 0.11 | 0.04 |
| Proportion of Variance | 0.87 | 0.06 | 0.04 | 0.02 | 0.01 |
| **Cumulative Variance** | 0.87 | 0.93 | 0.97 | 0.99 | 1.00 |
| **Model Fit Statistics** |  |  |  |  |  |
| Root Mean Square Residuals (RMSR) | 0.14 |  |  |  |  |
| Fit based upon off-diagonal values | 0.95 |  |  |  |  |

**Notes:** According to Kaiser’s criterion, acceptance is reserved for all Eigen > 1

**Appendix S3.** Correlation Matrix of Household Characteristics Psychological and Farm-level Variables

The correlations indicate milt associations across variables. The high correlation between age and residence time reflects structural realities in the Moroccan smallholder context, where older farmers are typically those with longer settlement and embeddedness in their communities. We intentionally retained both variables in the models because they capture distinct yet overlapping dimensions of contextual experience: age as a proxy for life‑cycle stage, and residence time as an indicator of local embeddedness and accumulated place‑based knowledge. To address potential irregularities, we employed a parsimonious backward stepwise model guided by the Akaike Information Criterion (AIC), which allowed us to assess whether either variable remained significant under alternative specifications. Although variance inflation factors (VIF) were not formally computed, heteroscedasticity‑robust standard errors were applied to mitigate bias in inference.

**Table 3. Pearson Correlation Matrix of Psychological, Socio-economic, and Farm-level Variables**

|  | Variable | 1 | 2 | 3 | 4 | 5 | 6 | 7 | 8 | 9 | 10 | 11 | 12 | 13 | 14 | 15 | 16 | 17 | 18 | 19 | 20 | 21 | 22 |
| --- | --- | --- | --- | --- | --- | --- | --- | --- | --- | --- | --- | --- | --- | --- | --- | --- | --- | --- | --- | --- | --- | --- | --- |
| 1 | **Concern Index** | - |  |  |  |  |  |  |  |  |  |  |  |  |  |  |  |  |  |  |  |  |  |
| 2 | **Openness** | .03 | - |  |  |  |  |  |  |  |  |  |  |  |  |  |  |  |  |  |  |  |  |
| 3 | **Conscientiousness** | -.08 | -.07 | - |  |  |  |  |  |  |  |  |  |  |  |  |  |  |  |  |  |  |  |
| 4 | **Extraversion** | -.06 | .08 | .27*** | - |  |  |  |  |  |  |  |  |  |  |  |  |  |  |  |  |  |  |
| 5 | **Agreeableness** | -.21*** | .10 | .12* | .37*** | - |  |  |  |  |  |  |  |  |  |  |  |  |  |  |  |  |  |
| 6 | **Emotional Stability** | -.60*** | .01 | .20*** | .24*** | .38*** | - |  |  |  |  |  |  |  |  |  |  |  |  |  |  |  |  |
| 7 | **Meta-Plasticity** | -.02 | .74*** | .13* | .74*** | .31*** | .17** | - |  |  |  |  |  |  |  |  |  |  |  |  |  |  |  |
| 8 | **Meta-Stability** | -.46*** | .03 | .52*** | .41*** | .76*** | .80*** | .30*** | - |  |  |  |  |  |  |  |  |  |  |  |  |  |  |
| 9 | **Structured Exploration**¹ | -.08 | -.73*** | .73*** | .13* | .02 | .13* | -.41*** | .33*** | - |  |  |  |  |  |  |  |  |  |  |  |  |  |
| 10 | **Social Harmony**² | -.39*** | .08 | .26*** | .71*** | .80*** | .72*** | .54*** | .89*** | .12* | - |  |  |  |  |  |  |  |  |  |  |  |  |
| 11 | **Age** | .01 | -.14** | -.11* | .00 | .01 | .08 | -.10 | .01 | .02 | .04 | - |  |  |  |  |  |  |  |  |  |  |  |
| 12 | **Education Level** | .22*** | .13* | .04 | .08 | -.03 | -.10 | .15** | -.06 | -.06 | -.02 | -.32*** | - |  |  |  |  |  |  |  |  |  |  |
| 13 | **Residence Time** | -.06 | -.12* | -.11* | -.04 | .01 | .11* | -.11* | .03 | .01 | .03 | .80*** | -.31*** | - |  |  |  |  |  |  |  |  |  |
| 14 | **Household Size** | .05 | .09 | .08 | -.05 | .08 | -.07 | .03 | .03 | -.01 | -.01 | .05 | -.15** | .06 | - |  |  |  |  |  |  |  |  |
| 15 | **Income Generation** | .04 | .01 | .06 | -.08 | -.02 | .03 | -.04 | .03 | .03 | -.03 | .15** | -.12* | .15** | .43*** | - |  |  |  |  |  |  |  |
| 16 | **Plain Topography** | .03 | .12* | -.02 | .02 | .00 | .00 | .10 | -.01 | -.09 | .01 | .09 | .10 | .07 | .02 | .10 | - |  |  |  |  |  |  |
| 17 | **High Fertility** | -.55*** | .11* | .14** | .08 | .25*** | .51*** | .12* | .46*** | .02 | .38*** | .01 | -.20*** | .01 | .04 | .06 | -.07 | - |  |  |  |  |  |
| 18 | **Land Size** | .04 | .14* | .06 | .05 | .02 | .05 | .13* | .06 | -.06 | .05 | -.02 | .05 | -.01 | .01 | -.01 | .06 | .02 | - |  |  |  |  |
| 19 | **Climate Exposure** | .36*** | .08 | .00 | .09 | .12* | -.23*** | .11* | -.06 | -.05 | .00 | .06 | .07 | .03 | .05 | -.01 | .04 | -.46*** | .02 | - |  |  |  |
| 20 | **No. Rooms** | -.02 | -.02 | .05 | .17** | .03 | .09 | .11 | .08 | .04 | .13* | .09 | .01 | .01 | .20*** | .19*** | -.01 | .13* | -.04 | -.04 | - |  |  |
| 21 | **Productive Assets** | -.12* | .12* | .17** | .09 | .09 | .13* | .14** | .17** | .04 | .14* | -.04 | -.02 | -.10 | .13* | .15** | .00 | .21*** | .07 | .01 | .37*** | - |  |
| 22 | **Livestock Assets** | -.25*** | .08 | .12* | -.01 | .01 | .17** | .05 | .14* | .03 | .07 | -.08 | -.08 | -.06 | .02 | .10 | -.04 | .30*** | -.04 | -.27*** | .09 | .22*** | - |

**Appendix S4.** Estimates of the Interaction of the Big Five Personality Traits with Affective Climate-Risk Perceived.

This table presents the OLS regression results for the direct association of the Big Five personality traits on the Affective climate-risk perception. Model [A] shows the base psychological model without controls (socio-economic variables). Model [B] includes the full suite of control variables. Model [C] presents the final, parsimonious model after a backward stepwise selection of controls.

| **Variable** | **Base Model** | **Full Controls** | **Parsimonious Model** |
| --- | --- | --- | --- |
| **Personality Traits** | **[A]** | **[B]** | **[C]** |
| Openness | 0.047  (0.050) | 0.065  (0.045) | 0.077*  (0.043) |
| Conscientiousness | -0.117  (0.151) | -0.080  (0.129) |  |
| Extraversion | 0.260***  (0.085) | 0.138*  (0.074) | 0.117*  (0.067) |
| Agreeableness | -0.055  (0.122) | -0.053  (0.105) |  |
| Emotional Stability | -1.257***  (0.085) | -0.849***  (0.084) | -0.875***  (0.078) |
| **Household Characteristics** |  |  |  |
| Age |  | 0.102  (0.075) | 0.074  (0.048) |
| Level of Education |  | 0.020**  (0.008) | 0.019***  (0.007) |
| Residence Time |  | -0.002  (0.004) |  |
| Household Size |  | 0.010  (0.015) |  |
| Number of Income Earners |  | 0.053*  (0.030) | 0.055**  (0.027) |
| Number of Rooms |  | -0.008  (0.026) |  |
| Climate Exposure Intensity |  | 0.112***  (0.022) | 0.108***  (0.021) |
| **Farm Characteristics & Assets** |  |  |  |
| Topography [Plain = 1] |  | -0.130*  (0.071) | -0.126*  (0.070) |
| Fertility [High = 1] |  | -0.520***  (0.102) | -0.531***  (0.099) |
| Land Size |  | 0.001  (0.001) |  |
| Productive Assets |  | -0.008  (0.018) |  |
| Livestock Assets |  | -0.046**  (0.023) | -0.051**  (0.023) |
| Constant | 3.291***  (0.694) | 2.077***  (0.612) | 1.629***  (0.340) |
| Observations | 331 | 331 | 331 |
| R-squared | 0.465 | 0.644 | 0.641 |
| Adjusted R-squared | 0.456 | 0.623 | 0.629 |
| F-statistic | 51.926*** | 30.584*** | 52.452*** |

***Notes:*** *Dependent variable is the Affective Climate Risk Perceived (denoting farmers worry). Robust standard errors are in parentheses. Significance levels: * p<0.1, ** p<0.05, *** p<0.01*

**Appendix S5.** Estimates of the link between Higher-order Meta-Trait Configuration and Perceived Affective Climate-Risk

This table presents the OLS regression results for the relationship between meta-traits and the level of affective climate-risk perceived by farmers. Model [A] shows the base psychological model without controls, including the non-linear term. Model [B] includes the full suite of controls. Model [C] presents the final, parsimonious model after stepwise selection.

| **Variable** | **Base Model** | **Full Controls** | **Parsimonious Model** |
| --- | --- | --- | --- |
| **Meta-Trait Configurations** | **[A]** | **[B]** | **[C]** |
| Meta-Stability | -0.674***  (0.118) | -0.397***  (0.105) | -0.381***  (0.057) |
| Meta-Plasticity | 0.263***  (0.066) | 0.162***  (0.056) | 0.163***  (0.047) |
| Structured Explore | 0.195***  (0.058) | 0.096*  (0.049) | 0.093*  (0.048) |
| Social Harmony | 0.107  (0.124) | 0.015  (0.106) |  |
| Social Harmony (Squared) | 0.095***  (0.029) | 0.043*  (0.025) | 0.044*  (0.024) |
| **Household Characteristics** |  |  |  |
| Age |  | 0.107  (0.080) |  |
| Level of Education |  | 0.021**  (0.008) | 0.018**  (0.008) |
| Residence Time |  | -0.004  (0.004) |  |
| Household Size |  | 0.030*  (0.015) | 0.034**  (0.014) |
| Number of Income Earners |  | 0.039  (0.032) |  |
| Number of Rooms |  | -0.023  (0.027) |  |
| Climate Exposure Intensity |  | 0.131***  (0.023) | 0.135***  (0.023) |
| **Farm Characteristics & Assets** |  |  |  |
| Topography [Plain = 1] |  | -0.131*  (0.076) | -0.109  (0.075) |
| Fertility [High = 1] |  | -0.610***  (0.107) | -0.618***  (0.105) |
| Land Size |  | 0.0005  (0.001) |  |
| Productive Assets |  | 0.001  (0.019) |  |
| Livestock Assets |  | -0.042*  (0.025) | -0.043*  (0.024) |
| Constant | -0.082  (0.052) | -0.364  (0.297) | -0.380*  (0.208) |
| Observations | 331 | 331 | 331 |
| R-squared | 0.355 | 0.597 | 0.591 |
| Adjusted R-squared | 0.344 | 0.573 | 0.577 |
| F-statistic | 32.933*** | 25.037*** | 42.418*** |

***Notes.*** *Dependent variable is the Affective Climate Risk Perceived (denoting farmers worry). Robust standard errors are in parentheses. Significance levels: * p<0.1, ** p<0.05, *** p<0.01*
